# The Digital Health Revolution and People with Disabilities: Perspective from the United States

**DOI:** 10.3390/ijerph17020381

**Published:** 2020-01-07

**Authors:** Mike Jones, Frank DeRuyter, John Morris

**Affiliations:** 1 Virginia C. Crawford Research Institute, Shepherd Center, Atlanta, GA 30309, USA; mike.jones@shepherd.org; 2 Department of Surgery, Duke University, Durham, NC 27708, USA; frank.deruyter@duke.edu

**Keywords:** disability, medical rehabilitation, mobile rehabilitation, mRehab, digital health, health disparities, chronic conditions, information and communication technology

## Abstract

This article serves as the introduction to this special issue on Mobile Health and Mobile Rehabilitation for People with Disabilities. Social, technological and policy trends are reviewed. Needs, opportunities and challenges for the emerging fields of mobile health (mHealth, aka eHealth) and mobile rehabilitation (mRehab) are discussed. Healthcare in the United States (U.S.) is at a critical juncture characterized by: (1) a growing need for healthcare and rehabilitation services; (2) maturing technological capabilities to support more effective and efficient health services; (3) evolving public policies designed, by turns, to contain cost and support new models of care; and (4) a growing need to ensure acceptance and usability of new health technologies by people with disabilities and chronic conditions, clinicians and health delivery systems. Discussion of demographic and population health data, healthcare service delivery and a public policy primarily focuses on the U.S. However, trends identified (aging populations, growing prevalence of chronic conditions and disability, labor shortages in healthcare) apply to most countries with advanced economies and others. Furthermore, technologies that enable mRehab (wearable sensors, in-home environmental monitors, cloud computing, artificial intelligence) transcend national boundaries. Remote and mobile healthcare delivery is needed and inevitable. Proactive engagement is critical to ensure acceptance and effectiveness for all stakeholders.

## 1. Introduction

Healthcare in the United States (U.S.) is undergoing a sea change, roiled by a political stormfront over efforts to improve access and manage costs of care delivery. Changing markets are exerting additional pressure on healthcare delivery models to engage consumers in managing more of their own care. Enabling this transformation has been the rapid development and use of emerging information and communication technologies (ICT). “Digital health” technology is beginning to shape how care is provided and received throughout the health ecosystem. In the U.S. and elsewhere, this means supporting the shift away from a “fee-for-service” model that pays healthcare providers based on the quantity of services delivered, toward a value-based care model focused on patient outcomes and overall health [1].

It also offers new possibilities for providing care to more people. As the World Health Organization notes, digital health (eHealth) will play “a unique and pivotal role in achieving universal health coverage” in many countries because “it extends the scope, transparency and accessibility of health services and health information, widening the population base capable of accessing the available health services and offering innovation and efficiency gains in the provision of health care” [2].

However, digital health (or eHealth), is still in the relatively early stages of development and adoption in the U.S. [3], as well as in other regions and countries, including Europe [4] and Australia [5]. While the digital transformation of healthcare creates new opportunities for access and participation by consumers and the potential for more efficient healthcare delivery, there are also concerns that people with disabilities and others with debilitating chronic health conditions may be left behind. Early evidence suggests that health disparities already exist and are likely to increase with the uptake of digital health technologies [6]. The “full potential of digital health technologies (ICT) to reach a large number of people with disabilities...and provide them with effective interventions has yet to be realized” [7].

We use the World Health Organization’s International Classification of Functioning, Disability and Health (ICF) to define disability [8]. The ICF focuses on three main domains: (1) individual body structure and functioning (e.g., mental, sensory, speech, movement function), (2) individual activities and participation (e.g., learning, communication, mobility, self-care), and (3) environmental factors that facilitate or impede social participation and environmental access (e.g., technology, natural and built environment, policy and attitudes). This structure synthesizes the “medical model” of disability as originating from disease, trauma, aging or other health conditions and the “social model” that sees disability as a socially created problem. Although the focus here is mainly on digital health and medical rehabilitation, it encompasses concerns relevant to both the medical and social components of disability.

This article summarizes several societal, technical, and policy trends in the U.S. that will influence the demand for and supply of available resources for medical rehabilitation. These trends include: (1) the aging of the U.S. population, (2) growth in the population of people with either permanent or temporary disabilities who require medical rehabilitation, (3) limitations in available resources, such as personnel and reimbursement for delivery of these services, (4) tightening of available labor supply to attend to increasing demand for health services, and (5) the emergence of new technologies that offer the potential to extend health services to more people and potentially enable improved and more timely care. Other important trends are the evolution of public policy and health insurance reimbursement policies designed to promote improved health outcomes. Although our focus is on healthcare in the United States, particularly with respect to healthcare reimbursement, the impact of these trends will be felt in many countries as the demand for medical rehabilitation and the gap in available resources to meet this demand continues to grow globally. These trends will contribute to a broader adoption of digital health solutions. If successfully executed, digital health could also reduce health disparities facing people with disabilities.

## 2. Discussion

### 2.1. Disability and Chronic Health

Estimates from the last U.S. Census of Population indicate that 56.7 million people, 18% of the population, were living with disability in 2010 [9]. Assuming an increase proportional to the expected 12.5% growth in the population, over 64 million people with disabilities will reside in the U.S. by 2025. Population trends suggest this number may be even higher as people with disability are living longer [10]. More people who experience a disabling health event (e.g., stroke, spinal cord injury, traumatic brain injury) are surviving and living longer. Survival rates in the first year post-injury have increased significantly in strokes [11,12], spinal cord injuries [13], and traumatic brain injuries [14]. In addition to living longer, people with disabilities are experiencing more chronic health conditions [15]. Over 80% of people with disabilities have one or more chronic conditions that compound the effects of disability on health and function [16]. For disabled adults, rates of physical inactivity are 120% higher [17], obesity 57% higher [18], smoking 47% higher [19], and hypertension 13% higher [20] than for nondisabled adults. People with disabilities of all ages have more than twice the incidence of diabetes [21], and rates of cardiovascular disease, the leading cause of death in the U.S., are three times higher [20,22].

Globally, it is estimated that over 1 billion people (approximately 15% of the population) have a disability of some type and degree of severity. Of this number, it is estimated that 110 to 190 million adults experience significant difficulties in functioning, and 93 million children (1 in 20 of those under 15 years of age) live with moderate or severe disability. The number of people with a disability is expected to increase as populations age and the number of people with chronic health conditions increase. Notably, disability rates tend to be higher in lower-income countries due to environmental and other factors, including more limited access to health care services [23].

### 2.2. Growing Demand for Health and Rehabilitation Services

For decades, healthcare spending in the U.S. grew robustly, averaging 9% annually. Over the past 10 years, annual growth slowed to 4%, due to the Great Recession, payment and delivery system reforms under the Patient Protection and Affordable Care Act (ACA), increased use of generic drugs, and lower Medicare payments for services, including medical rehabilitation. Future healthcare spending is expected to grow on average 5.5% annually through at least 2027, outpacing average growth in gross domestic product (GDP) [24], driven by growth in volume and intensity of healthcare services, and the shift from private health insurance to Medicare.

Several societal trends are converging that will increase the demand for and expected cost of rehabilitation services. An aging general population means more older people are likely to experience disabling conditions requiring medical rehabilitation. The percentage of Americans aged 65 or older is estimated to increase from 13.0 percent of the population in 2010 to 16.1 percent in 2020, 19.3 percent in 2030, and 20 percent by 2040. The total number of people in the U.S. aged 65 or older will more than double from 40.23 million in 2010 to 81.24 million in 2040 [25]. Riding the crest of this trend is the baby boomer generation—people in the United States and other advanced economies born between 1946 and 1964. Boomers are living longer than previous generations, but also exhibit important health and behavioral differences. While less likely to smoke, they have a 55% higher prevalence of diabetes, 25% higher prevalence of obesity, and 9% lower prevalence of “very good” to “excellent” self-rated health [26]. The long-term consequence is likely to be more years spent with chronic health conditions and disability by a larger percentage of the population, which will dramatically increase the demand for health and rehabilitation services over the next mid-century.

Aging of the population represents a substantial challenge in many other countries, especially economically advanced countries. The United Nations estimates that the percentage of the global population of people age 65 and older is at 9.3% in 2020, up from 8.2% in 2015 and 7.6% in 2010. This figure is predicted to rise to 11.7% in 2030 and 15.9% in 2050. In more developed countries the population 65 years of age and older is estimated to be 19.3% in 2020 and 22.9% in 2030 [27].

In anticipation of this growing demand, several changes in reimbursement for medical rehabilitation in the U.S. have been enacted over the past 20+ years and more changes are on the horizon. First, inpatient rehabilitation payment under Medicare (the federal government health insurance program and the largest funder of medical rehabilitation services in the U.S.) underwent a major transformation in 1997 with legislation mandating a transition from fee-for-service to prospective payment based on diagnosis. This legislation also mandated a shift in patient mix to include more severely impaired/medically complex patients, so that by 2012 at least 60% of patients admitted for inpatient rehab were required to be of high-severity and/or complexity. These mandates resulted in a decrease in length-of-stay (LOS) for inpatient rehabilitation from a median of 20 days in 1994 to 12 days in 2001. Since 2004, the median LOS has remained stable (12.9 days), despite a steady increase in severity/complexity of patients admitted for inpatient rehabilitation [28,29].

Second, perhaps the most significant change for Medicare reimbursement of rehabilitation services is the proposed unified prospective payment system (PPS) for post-acute care (PAC). The Improving Post-Acute Care Transformation (IMPACT) Act of 2014 is a mandated development of a single PAC PPS, with payment based on a patient’s clinical characteristics instead of care setting and therapy use. Using 2013 reference-year data (comprising over 5.3 million episodes of care), the U.S. Centers for Medicare and Medicaid Services (CMS) is establishing unified payment for rehabilitation services regardless of the venue of care (inpatient rehabilitation facilities, skilled nursing facilities, long-term care hospitals, home health agencies). Expected to be phased in over a 3-year period beginning in 2021, the PAC PPS will have sweeping consequences for venues of care. There is no question this reform will hasten the transition to outpatient and home-based rehabilitation and is likely to force the closure of many inpatient rehabilitation programs, particularly those in under-served areas [30].

Third, “rehabilitative and habilitative services and devices” were incorporated as one of 10 “essential benefits” under the Affordable Care Act (ACA) in 2010. These services and devices are defined as those that “help people with injuries, disabilities, or chronic conditions gain or recover mental and physical skills.” The ACA requires that health insurance plans sold through the health insurance exchange include habilitative and rehabilitative benefits, however, the scope of benefits is determined by each state. States have considerable latitude in determining how plans administer these benefits and the out-of-pocket expenses for beneficiaries. As a result, the actual benefits (e.g., number of outpatient visits/year) vary significantly from state to state. Moreover, benefit levels do not vary based on severity of condition [31]. One consequence is that the defined essential benefit (e.g., 30 outpatient visits per year) has become the default standard regardless of patient need. While coverage offered by these plans meets the essential-benefits requirements, out-of-pocket expenses may be substantial. Thus, another consequence of these plans is that many insured individuals are foregoing preventive health services (including outpatient rehabilitation) because of higher out-of-pocket costs.

Finally, there is a significant gap in the available workforce capacity in the U.S. for delivery of medical rehabilitation services to address growing demand. According to the U.S. Bureau of Labor Statistics, the demand for rehabilitation physicians, allied health professionals, and specialty nursing in medical rehabilitation is expected to grow by 17%–28% from 2016 to 2026 [32]. Thus, disparities in healthcare access by people with disabilities are likely to be further exacerbated by the growing gap between the demand for and the supply of health and rehabilitation services.

### 2.3. Inefficiencies in Our Current Approach to Medical Rehabilitation

Although the benefit of medical rehabilitation is well established, inefficiencies in the current system of care in the U.S. are also well documented. For example, a CDC survey found that only 31% of stroke survivors received outpatient rehabilitation [33], significantly fewer than recommended by clinical guidelines [34]. Even when offered, these services are often unstructured, difficult to obtain, and not driven by progressive goals to improve function or reduce disability [35].

One response to reimbursement pressures and the gap between supply and demand for rehabilitation services has been a shift from inpatient to outpatient venues of care, which will continue with enactment of the PAC PPS in 2021. In addition to lower overall costs, outpatient care offers the potential advantage of tailoring therapies to the home and community setting where the patient resides. However, the advantages of outpatient care are offset by several challenges to effective service delivery. Frequent problems faced by clinicians delivering outpatient care for patients with chronic conditions include measuring gains and losses of daily functioning over time, assessing the effects of timing and dose of interventions related to physical functioning (e.g., home exercise), assessing adherence to instructions for exercise, providing adequate feedback about performance of exercises, and being able to update instructions more frequently than only at the time of periodic outpatient visits [36].

A related problem in outpatient therapy is the disconnect between demonstrated functional capacity in the clinic and improved functional performance in daily life. For example, Waddell and colleagues [37] measured upper limb (UL) functional capacity of stroke survivors using standardized assessments conducted during outpatient clinic visits, and measured UL performance by having patients wear accelerometers on each wrist at home. Results showed a high degree of variability in UL capacity and performance, with a small minority of patients demonstrating concordance between improved capacity and improved performance. The results challenge a common assumption among therapists that improvements in capacity observed in the clinic translate into improved performance in daily life [38].

### 2.4. Digital Health Technologies to Address Problems of Healthcare Access and Affordability

The U.S. National Institutes of Health (NIH) Research Plan on Rehabilitation released in 2017 recognizes the potential of information and communication technologies (ICT) to revolutionize medical rehabilitation with mobile applications, including: “symptom monitoring, real-time data capture, real-time access to information about (patients) navigating the community, social connectedness through peer-to-peer support, and bidirectional communication” [2]. The potential of mobile rehabilitation, or mRehab, is also recognized in a recent report on Emerging Technologies to Support an Aging Population published by the U.S. National Science & Technology Council [39]. The Report calls for the development of systems to enable home therapy in order “to minimize cost, maximize access, and maximize the amount of time that can be spent pursuing rehabilitation activities.” Recommended applications include gamification to improve adherence and engagement, development of affordable devices to promote intensive practice, use of smart-home technologies for rehabilitation, including monitoring of improvement, and development of algorithms that “combine data from movement and activity sensors to provide increased accuracy in assessment of individual well-being. These may include use of artificial intelligence and should provide useful information to users, healthcare providers, and caregivers” [39].

With continued growth in the ICT field, development of digital health applications can significantly broaden rehabilitation access for people with disabilities. Major ICT companies, and numerous health-specific startups, have introduced mobile health solutions and whole platforms (e.g., Apple’s HealthKit, Google Fit, Samsung Health) to support the capture, storage and reporting of user health, fitness and activity data. Specialized vendors offer secure, cloud data collection, storage and visualization tools for self-report and sensor-derived patient data (e.g., Datu), and management platforms for clinicians to support home- and community-based exercise and other rehabilitation interventions (e.g., Pt Pal, FlintRehab).

If executed successfully, mRehab strategies could address key challenges of access and affordability. As the NIH Research Plan notes, “the use of ICT eliminates distance barriers and can make rehabilitation and healthcare services available to people who have limited access to transportation and other access issues” [2]. ICT-enabled home rehabilitation interventions between outpatient visits offer the potential to fill gaps that exist in patient care by (1) prescribing interventions/instructions to the patient and caregiver, (2) gathering timely data on patient status instead of relying on imprecise recall during clinic visits, (3) presenting data to the patient and clinician in a timely manner, and (4) updating prescribed in-home therapy and recommendations.

Effective mRehab innovations may also address challenges to effective and efficient delivery of outpatient rehabilitation by increasing patient motivation and adherence to recommended therapy regimens, improving patient engagement, and permitting more rapid progression between clinic visits. For example, Flint Rehab Devices, LLC has already demonstrated dramatic increases in patient engagement in home-based exercises using their inexpensive, simple-to-use, sensor-enhanced rehab devices [40]. They found that patients far exceeded the number of recommended exercise “reps” using gamified exercises with their FitMi device [41]. mRehab interventions using ICT may also afford clinicians insight into patient functioning at home, resulting in greater efficiency in choice of treatments (e.g., change treatments based on patient (non)adherence or (lack of) progress). This may also improve concordance between patients’ observed capacity in the clinic and functional performance at home.

In line with the push toward location-neutral payment for post-acute rehabilitation services (PAC PPS), mRehab presents the opportunity for rehabilitation providers to incorporate ICT into alternative models of intervention delivery and management not previously available. Providers will be able to maximize the impact of limited post-acute benefits (e.g., number of outpatient visits) by providing a technological bridge to support home-based therapy between clinic visits. This way, outpatient visits can be spread out over time while still providing important support to the patient to ensure progress is made between visits. However, several important questions must be answered before successful adoption of alternative models of care: (1) does mRehab technology improve and maintain patient adherence and engagement; (2) do clinicians’ insights into patient functioning at home and in the community improve success in achieving targeted therapy goals; (3) does mRehab technology contribute to greater patient autonomy and control in intervention delivery and effectiveness; (4) can insights from the data collected using digital health technology be used to improve efficiency of service delivery (e.g., determine optimal follow-up intervals, generate algorithms to progress therapy based on patient performance, improve concordance between functional capacity and performance); and (5) do alternative models using mRehab deliver greater return on investment of limited healthcare dollars?

### 2.5. Challenges to Adoption of mRehab

Assuming the questions above can be answered affirmatively, there remain issues of acceptance by consumers, rehabilitation providers, and healthcare provider organizations. Issues of acceptance for consumers include familiarity and comfort with use of technology, human-technology interface limitations (accessibility and usability) for people with disabilities, concerns about privacy and intrusiveness, and loss or diminished contact with the clinical service provider (less personalized care) [42].

Potential benefits include expanded access to services, enhanced empowerment for one’s own health outcomes, and the opportunity to progress at the desired pace. The ability to identify different profiles of patients with respect to adherence, engagement, and progress suggests an opportunity to create a taxonomy of behavioral characteristics, models of treatment delivery and intervention strategies (e.g., use of gamification, social support, other “nudges” to maintain adherence).

Potential barriers to acceptance by clinicians include: familiarity with technology, complexity and time requirements for use of the technology, practice needed to become proficient, changes required in clinical workflow to incorporate the approach, and the need for practice standards and evidence of effectiveness to govern and justify use of the approach. The issue of reimbursement for the non-hands-on time required by clinicians to support an mRehab system (e.g., review and respond to patient data or inquiries) is also a key concern. On this front, a recent final rule by CMS authorized the addition of three new Medicare billing codes for “Remote Patient Monitoring” (RPM) [43]. The codes cover activities related to RPM of physiologic parameters (e.g., weight, blood pressure, pulse oximetry, respiratory flow rate), and address (1) initial set-up and patient education, (2) review of daily patient data or programmed alerts, and (3) at least 20 min of interactive communication with the patient per month. Digital health advocates recently asked CMS to clarify the technologies covered under the new RPM codes, recommending coverage for smartphone applications, Fitbits, and artificial intelligence messaging. CMS has not offered specifics on what technology qualifies but does plan to issue guidance on these issues.

“Institutional inertia” on the part of provider organizations will also be an ongoing challenge. Factors that affect adoption include lack of evidence-based interventions, need for investment of resources (technology, staff expertise) to implement new practices, required systems changes in models of care and clinical workflows, and lack of verified return on investment. With respect to the shifting landscape of healthcare reimbursement, concepts like value-based care, bundled-payment, and population health management may not be at the forefront in the current political environment, but there can be no question that healthcare providers will be expected to do more with less, and digital health technologies may offer part of the solution.

Despite the myriad challenges, there is evidence of growing interest in mRehab interventions by rehabilitation providers. Results from our 2019 survey of over 500 rehabilitation practitioners (medical, nursing, allied health) in the U.S., published in this issue [44], reveals wide recognition of the need for additional therapeutic interventions after discharge from inpatient rehabilitation and additional rehabilitation intervention between outpatient visits. However, less than half of respondents were currently comfortable with integrating mRehab into their practice and less than a quarter believe they are knowledgeable about technologies that could be used for mRehab. The potential benefit is recognized but providers need additional knowledge and support to comfortably incorporate these approaches into practice—understandable given the emerging state of the field.

This disconnect between clinicians’ recognition of the need for digital health and their willingness to integrate it into their practice and confidence in their knowledge of digital health solutions is widespread. A 2016 survey of physicians in the U.S. reveals that a majority sees mastering digital health technology as necessary to stay current with healthcare practice. Yet, most respondents reported being concerned about potential liability, reimbursement, technical problems and patient privacy [45]. Similarly, a survey of physicians in Europe documented great concern over new public health challenges like the aging of the population and rise of infectious disease, and high expectations for digitalization of patient data, wearable technology and mobile apps, and telehealth solutions. These same physicians reported lower than expected rates of adoption of eHealth solutions like electronic prescribing of medications and telehealth [46]. Additionally, these physicians cited patient privacy and data security as major concerns. A scoping review of the literature on physician attitudes toward eHealth conducted by Canadian researchers identified technology design, training, liability and patient privacy as key issues [47]. Tension between interest and optimism for digital health and limited knowledge of digital health technology has been identified elsewhere, including Somalia [48] and Iran [49].

## 3. Conclusions

The transformation of medical rehabilitation is evident. Although the benefits of rehabilitation are well established, inefficiencies in the current system of care are also clearly documented. These inefficiencies are influenced by the growing demand for services, continued changes in reimbursement streams in the United States to contain cost, pivoting service delivery models, and increasing provider shortages. Today’s priorities in medical rehabilitation are containing costs, improving access, and increasing the amount of time spent on actual rehabilitation.

It has been suggested that the transformation toward the greater use of digital health technologies will lead to better outcomes, greater value in care, better patient experiences, and more empowered rehabilitation stakeholders. While initial anecdotal evidence appears to support this contention, these changes will not be realized without challenges.

Challenges to adoption include the rate by which patients and providers are overwhelmed by the increasing number of disruptive technologies introduced into practice. There continues to be too much focus on the technology as opposed to the human element. This is often observed with the inconsistent coaching provided to patients when sensors are included in behavioral change platforms. Opportunity exists for further research to identify potential barriers and facilitators of adoption of digital health in rehabilitation.

In order to ensure acceptance by patients and providers, meaningful information about successful mRehab applications is paramount. Efforts are needed to develop, deploy and evaluate efficacy of innovative technological solutions. Knowledge translation must address both effectiveness and user acceptance of new mRehab applications. Additionally, issues related to adoption of mRehab interventions and technologies by provider organizations cannot be ignored. Ultimately, those who control budgets must be convinced that adoption of mRehab strategies into clinical practice provides a positive return on investment with respect to safe and effective service delivery, improved outcomes and patient experience, and reasonable risk for the enterprise.

Remote and mobile healthcare delivery is needed and inevitable. Demographic, population health trends and labor force trends (aging populations, growing prevalence of chronic conditions and disability, labor shortages in healthcare) in countries with advanced economies, and other countries, will continue to exert ever greater pressure on existing healthcare resources, creating ever more urgency for technology-based healthcare service innovations. Technologies that enable mRehab (wearable sensors, in-home environmental monitors, cloud computing, artificial intelligence) are now sufficiently mature to support widespread implementation of new service delivery solutions. Proactive engagement by all stakeholders will be critical to ensure acceptance and effectiveness.
